# Ahead of others in the authorship order: names with middle initials appear earlier in author lists of academic articles in psychology

**DOI:** 10.3389/fpsyg.2015.00469

**Published:** 2015-04-21

**Authors:** Eric R. Igou, Wijnand A. P. van Tilburg

**Affiliations:** ^1^SOCO-UL Lab, Department of Psychology, University of LimerickCastletroy, Ireland; ^2^Centre for Research on Self and Identity, Psychology, University of SouthamptonSouthampton, UK

**Keywords:** names, middle initials, authorship order, publications, individual differences

## Abstract

Middle name initials are often used by people in contexts where intellectual performance matters. Given this association, middle initials in people’s names indicate intellectual capacity and performance (Van Tilburg and Igou, 2014). In the current research, we examined whether middle initials are associated with a typical academic indicator of intellectual performance: authorship order of journal articles. In psychology, authorship early in the author list of an article should correspond with greater contribution to this intellectual endeavor compared to authorship appearing later in the author list. Given that middle initials indicate intellectual capacity and performance, we investigated whether there would be a positive relationship between middle initials in author names and early (vs. late) appearance of names in author lists of academic journal articles in psychology. In two studies, we examined the relationship between amount of authors’ middle initials and authorship order. Study 1 used a sample of 678 articles from social psychology journals published in the years 2006 and 2007. Study 2 used a sample of 696 articles from journals of multiple sub-disciplines in psychology published in the years from 1970 to 2013. Middle initials in author names were overrepresented early (vs. late) in author lists. We discuss implications of our findings for academic decisions on authorship orders, potential avenues of further investigation, and applications.

## Introduction

### Names Matter

Research on the psychology of names has documented associations of names with judgments and behaviors of people. For example, research on the name letter effect (Nuttin, 1985, 1987; Kitayama and Karasawa, 1997; Koole et al., 2001; Pelham et al., 2002) has documented that through the relatively positive evaluation of letters of one’s name, a person is likely to positively evaluate targets that include these letters. These effects of name characteristics are, in most cases, non-conscious (e.g., Stieger and Burger, 2013; Stieger et al., 2014), and have been documented in lab settings and for real life preferences and decisions, although generality and reliability of such name effects and their underlying processes are debated (e.g., Pelham and Carvallo, 2011; Simonsohn, 2011a,b,c; Polman et al., 2013; Howard and Kerin, 2014). Besides appreciating the letters of one’s own name, the meaning of names can affect people. For example, Christenfeld et al. (1999) reported that people with initials that spell words with positive meaning live longer compared to those whose initials form words with negative meaning. Recently, it has been shown that unpopular first names lead to more interpersonal neglect (e.g., in online dating) than more popular first names (Gebauer et al., 2012). Further, the alphabetical position of the first letter of one’s childhood surname seems to be associated with the speed with which people acquire products as adults (e.g., Carlson and Conrad, 2011). Name effects have also been reported within academia. For example, authors in economics with surnames early in the alphabet have better reputations than other authors (Efthyvoulou, 2008), and seem to have greater chances of winning the Nobel Prize (Einav and Yariv, 2006). In sum, research has documented various associations between names and judgments and decisions in many domains of life, including academia.

Our current research examines the relationship between middle name initials of academics and order of names in author lists of academic journal articles in psychology. We will first report the recent research on the middle initial effect and then explain the rationale for the current research.

### Middle Names Matter

Van Tilburg and Igou (2014) recently documented that the presence of middle name initials leads to more favorable inferences about people’s status, their intellectual capacity and their performance. We argue that people associate middle initials with formal situations where intellectual capacity and performance matter (e.g., a doctor’s office, a lawyer’s letter head, an academic certificate). Based on this association, middle initials serve as symbolic representatives of intellectual capacity and performance. People then use middle initials in names of others as a cue to infer their intellectual capacity and performance.

The middle initial effect has been found, for example, with regard to writing performances of fictitious authors. Specifically, a scientific text was evaluated more positively when the alleged author’s name included middle initials than when the author’s name did not include middle initials (Van Tilburg and Igou, 2014; Studies 1 and 5), reflecting greater perceived intellectual performance when author names included middle initials than when their names did not include middle initials. Further, middle initial effects emerged for judgments and decisions that were related to intellectual performance, but less so for judgments and decisions that seemed unrelated to intellectual performances (e.g., athletic competitions; Study 3).

If middle initials positively affect perceived intellectual capacity and performance, then it is possible that they have an impact in an area where intellectual performance is crucial: academia. Academic performance is in part measured by scholars’ publication record, for example, the topics that academics covered, the quantity of their publications, the impact of journals that they publish in, citations of their work, and whether or not someone appears at particular symbolically important positions in the authorship order. We focus here on the last point: the authorship order of academic journal articles in psychology.

In psychology, academics are generally asked and trained to consider the guidelines of the American Psychological Association (APA) regarding author order. Specifically, for many years APA manuals (e.g., American Psychology Association [APA], 2010) have outlined that the author byline should be indicative of the authors’ contribution. Authors early in the byline are usually associated with the greater contribution. We focus on psychology because guidelines regarding authorship order do not unequivocally apply to all academic domains (e.g., Einav and Yariv, 2006). Of course, we don’t deny that there are deviations from this norm within psychology, and that some people rightfully or wrongfully question the norm altogether (e.g., Fine and Kurdek, 1993; Thompson, 1994). However, the norm to base authorship order on contribution exists and affects decisions about bylines of articles. We suggest that this is where middle initials may operate. Given that middle initials influence perceived intellectual capacity and performance, we examined whether middle initials in author names are associated with authorship order, namely that authors with middle initials are more often listed early (vs. late) in the authorship order of academic journal articles in psychology.

We examined the association between middle initials and authorship order in academic journal articles in psychology. Our previous research (Van Tilburg and Igou, 2014) demonstrates that middle initials in names positively influence perceived intellectual capacity and performance. According to APA guidelines, contribution should be reflected in authorship order: the greater the contribution, the earlier the listing in the byline. Given that middle initials lead to inferences about intellectual capacity and performance and that authorship order symbolizes intellectual performance, we reasoned that there might be a positive relationship between middle initials and early (vs. late) positions in author lists. We conducted two studies using publication records of academic psychology articles to examine this relationship. Study 1 included a sample of 678 articles from social psychology journals published in the years 2006 and 2007. Study 2 included a sample of 696 articles from journals of multiple sub-disciplines in psychology published in the years from 1970 to 2013. We expected that authors with many middle initials appear earlier in author lists than authors with fewer middle initials, and that author names with at least one middle initial appear earlier in author lists than authors without any middle initial.

The goal of the research was to examine the relationship between middle initials and authorship order manifested in publication records. We wish to note here that the correlational framework did not test causality. Further, given that we used publication records, our studies did not directly test the impact of specific processes such as inferences, judgments and decisions that may play a role during the research and publication process.

## Study 1

In the first study, we examined author names of published articles from three major social psychology journals^1^ for the years 2006 and 2007. We expected that authors with many middle initials would appear earlier in author lists than authors with few or no middle initials (‘many vs. fewer’), and that authors with one or more middle initials would appear earlier in author lists than authors with no middle initial (‘at least one vs. none’).

### Method

The content of three prominent social psychology journals during 2006 and 2007 was entered in a nested data file. Specifically, we entered 678 articles with 2,123 author names. These data hence contained two levels: the higher level consisted of data pertaining to *articles* and the lower level consisted of data pertaining to the *authors* of each of these articles. Specifically, this nested data file was organized by articles as higher level variables, whereas amount of middle initials and position of each author name were lower level variables. The dataset contained the names of the authors, the amount of middle initials, the author position, the journal, the journals’ impact factors, the total amount of authors on each paper, the gender of the authors, and the year of publication.

### Results and Discussion

To examine whether more middle initials in author names are associated with an earlier author position, we conducted two types of multilevel analyses of increasing complexity. The primary model contained the article as higher level random variable, and amount of middle initials as fixed lower level predictor of positions in the authorship order (*many versus fewer*). In a secondary multilevel model we included a random effect of the articles’ total amount of authors, and its interaction with the amount of middle initials of authors, given the likelihood of considerable variation in the average author position across articles. In this subsidiary model, we also added year of publication (dummy coded: 2006 = 0, 2007 = 1), journal impact factor, and author gender as fixed predictors. We repeated both analyses (primary and secondary) after dichotomizing the *amount* of middle initials into the *presence* of middle initials (*one or more versus none*). In addition, we repeated the resultant four analyses with only *multi-authored articles*, thus examining more strictly the relationship between middle initials and authorship order.

#### ‘Many versus Fewer’ for all Articles

Consistent with the prediction, the primary analysis revealed a negative association between the amount of authors’ middle initials and their positions in the author list, τ = -0.205, *S*_e_ = 0.057, *t*(2076.844) = 3.580, *p* < 0.001, indicating that authors appear earlier in articles’ author lists when they have many versus fewer middle initials. This middle initial effect remained significant when the covariates were added in the secondary analysis, τ = -0.243, *S*_e_ = 0.056, *t*(16267.62) = 4.309, *p* < 0.001. Among these covariates, the year of publication did not yield a significant association with authorship position, τ = 0.105, *S*_e_ = 0.065, *t*(1342.375) = 1.613, *p* = 0.107, and neither did gender, τ = -0.080, *S*_e_ = 0.057, *t*(2107.445) = 1.405, *p* = 0.160. Interestingly, the journals’ impact factor was positively associated with the author position, τ = 0.053, *S*_e_ = 0.023, *t*(1317.070) = 2.264, *p* = 0.024. Given that we examined only three journals, however, it is likely that this effect stems from differences in the average amount of authors who published in these journals. The amount of article authors yielded a significant association with authorship position, γ = 0.012, *S*_e_ = 0.002, *Z* = 6.822, *p* < 0.001. Also the interaction between amount of authors of the articles and amount of middle initials was significant, γ = 0.107, *S*_e_ = 0.003, *Z* = 4.196, *p* < 0.001. This indicates that the middle initial effect was stable across different amounts of authors.

#### ‘At Least One versus None’ for all Articles

**FIGURE 1:**
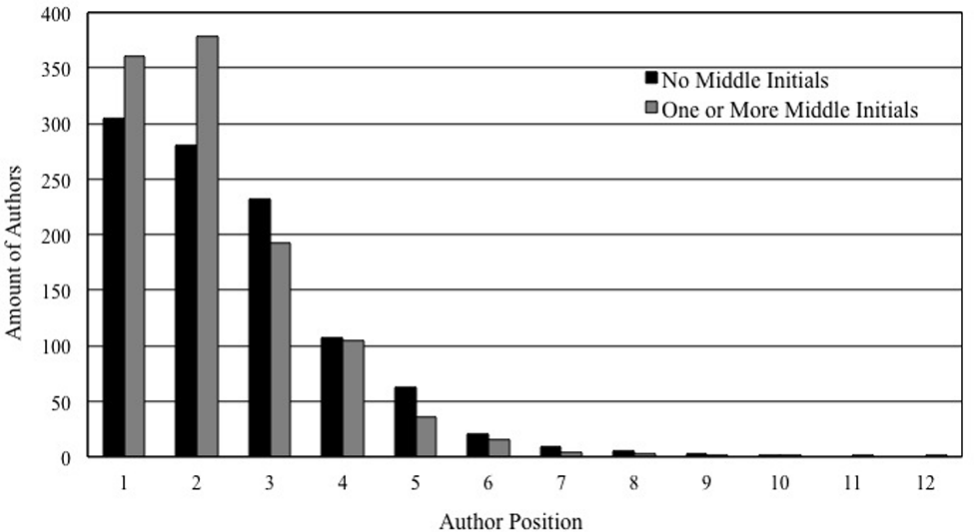
**The amount of authors for each author position based on the disaggregated data (Study 1)**.

Approximately half of the authors had no middle initials (48.2%). We therefore repeated the primary and secondary analyses after dichotomizing the amounts of middle initials (*one or more* versus* none*). Consistent with the previous results, the average position of authors with middle initials was significantly earlier in author lists (*M* = 2.26, SD = 1.34) than author names without middle initials (*M* = 2.51, SD = 1.49), τ = -0.228, *S*_e_ = 0.062, *t*(2062.376) = 3.690, *p* < 0.001 (for each author position see **Figure 1**). The association between middle initials and author positions remained significant when the covariates were added in the secondary analysis, τ = -0.272, *S*_e_ = 0.060, *t*(1786.360) = 4.505, *p* < 0.001. In this subsidiary analysis, the year of publication did not yield a significant association with authorship position, τ = 0.105, *S*_e_ = 0.065, *t*(1344.776) = 1.601, *p* = 0.110, and neither did gender, τ = -0.080, *S*_e_ = 0.057, *t*(2108.580) = 1.403, *p* = 0.61. The journals’ impact factor was positively associated with the author position, τ = 0.055, *S*_e_ = 0.023, *t*(1320.200) = 2.343, *p* = 0.019. The amount of article authors yielded a significant association with authorship position, γ = 0.012, *S*_e_ = 0.002, *Z* = 6.797, *p* < 0.001, as did the interaction between amount of authors on articles and presence of middle initials, γ = 0.012, *S*_e_ = 0.003, *Z* = 4.326, *p* < 0.001.

#### ‘Many versus Fewer’ for Multi-Authored Articles

Also after excluding single-authored articles from consideration, we observed an association between middle initial amounts and author positions, τ = -0.203, *S*_e_ = 0.057, *t*(2042.377) = 3.523, *p* < 0.001. This effect remained significant when adding the covariates in the secondary analysis, τ = -0.239, *S*_e_ = 0.057, *t*(1594.354) = 4.198, *p* < 0.001. Among these covariates, the year of publication did not yield a significant association with authorship position, τ = 0.103, *S*_e_ = 0.066, *t*(1311.828) = 1.562, *p* = 0.118, and neither did gender, τ = -0.081, *S*_e_ = 0.058, *t*(2077.643) = 1.402, *p* = 0.161. As before, the journals’ impact factor was positively associated with the author position, τ = 0.050, *S*_e_ = 0.024, *t*(1291.534) = 2.106, *p* = 0.035. The amount of article authors yielded a significant association with authorship position, γ = 0.012, *S*_e_ = 0.002, *Z* = 6.737, *p* < 0.001, as did the interaction between amount of authors of the articles and amount of middle initials, γ = 0.010, *S*_e_ = 0.003, *Z* = 4.149, *p* < 0.001.

#### ‘At Least One versus None’ for Multi-Authored Articles

Also for the multi-authored articles we conducted a similar multilevel analysis after recoding the amounts of middle initials into one group of authors with no middle initials, and one group of authors with one or more middle initials. Consistent with the results of our previous analyses, average positions of author names with middle initials were significantly earlier in author lists (*M* = 2.27, SD = 1.34) than author names without one or more middle initials (*M* = 2.52, SD = 1.49), τ = -0.226, *S*_e_ = 0.062, *t*(2027.572) = 3.611, *p* < 0.001. As before, the effect remained significant, when we controlled the other predictors in the secondary model, τ = -0.267, *S*_e_ = 0.061, *t*(1752.360) = 4.374, *p* < 0.001. The year of publication did not yield a significant association with authorship position, τ = 0.102, *S*_e_ = 0.066, *t*(1313.968) = 1.549, *p* = 0.122, and neither did gender, τ = -0.080, *S*_e_ = 0.058, *t*(2078.693) = 1.399, *p* = 0.162. Again, the journals’ impact factor was positively associated with the author position, τ = 0.052, *S*_e_ = 0.024, *t*(1294.493) = 2.184, *p* = 0.029. The amount of article authors yielded a significant association with authorship position, γ = 0.011, *S*_e_ = 0.002, *Z* = 6.713, *p* < 0.001, as did the interaction between amount of authors of the articles and presence of middle initials, γ = 0.012, *S*_e_ = 0.003, *Z* = 4.281, *p* < 0.001.

#### Omissions of Middle Initials?

It could be argued that authors responsible for the submission of an article – in most cases the first author – may at times accidently omit the middle initials of the co-authors, leading to the observed association between displayed middle initials and authorship order. We (authors and two assistants blind to the specific hypothesis) checked the data file regarding possible omissions of middle initials using two different sampling methods. For the first check, we took a random sample of 30 articles from the data file to check for these authors’ names in author lists of other publications and in other sources (e.g., webpages). Although the omission hypothesis seems plausible, we did not find any omissions in this data set. For the second check, we selected every fourth (i.e., 25%) of all listed articles in the data set and checked for omissions of middle initials by using additional published work of the authors and websites. We found 19 omissions in the data set of 170 articles, with five middle initial omissions at the first and at the second author positions, four omissions at the third author position, three at the forth author position, and one each at the fifth and sixth author positions. Given that over half of the omissions relate to authors early in the author list (first and second author), for which we document an *over*representation of middle initials, middle initial omissions improbably account for the observed association between middle initials and authorship order. Based on these results, the omission hypothesis seems unlikely to explain the results of Study 1^2^.

The results of Study 1 document that middle initials in author names are associated with earlier appearance in author list of social psychology journal articles in the years 2006 and 2007. Eight different statistical models reliably revealed the association between middle initials and authorship order: with or without covariates, including middle initials as continuous or dichotomous variable, and analyzing all articles versus only multi-authored ones.

## Study 2

In Study 1, we found that the amount of middle initials present in author names correlated negatively with their position in the author list. Perhaps, people born in the 1950s and 1960s may have been less likely to receive middle names than people in the following years. More senior academics, who possibly appear later in the authorship order, may have been born during this time and the results of the previous study may therefore reflect a cohort effect. To rule out this explanation and to examine the robustness of the association between middle initials and authorship order, we conducted a second study. Specifically, we examined the potential effect of cohorts and whether middle initial effects could also be observed for psychology journals from other areas than social psychology. The crucial hypotheses and statistical tests were identical to those of Study 1.

### Method

We examined authors from academic articles in eight prominent psychology journals^3^, excluding social psychology journals (cf. Study 1) from 1970 through 2013. Specifically, we entered 696 articles with 1,737 author names. We sampled research articles from the first and last issue of each year, and, where possible, the third article of each issue. If a journal only had two articles in an issue, we selected the second article.

As in Study 1, two levels of data were present: the higher level consisted of data pertaining to articles (publication year, journal, impact factor, number of authors) and the lower level consisted of data pertaining to the authors of each of these articles (middle initials, author position, gender).

### Results and Discussion

As in Study 1, we conducted two types of multilevel analyses. The primary model contained the article as higher level random variable, and the amount of middle initials as fixed lower level predictor of positions in the author list. The secondary model added a random effect of the articles’ total amount of authors, its interaction with the amount of middle initials of authors, the year of publication (1970 = 0 as reference), journal impact factor, and author gender as fixed predictors. We then repeated these analyses after dichotomizing the amount of middle initials into the presence of middle initials. First, we analyzed the association between middle initials and authorship order across all articles including single-authored articles and afterward we analyzed this association across all articles excluding single-authored articles.

#### ‘Many versus Fewer’ for all Articles

The first analysis revealed the predicted negative association between authors’ amount of middle initials and their position in the author list, τ = -0.244, *S*_e_ = 0.061, *t*(1681.623) = 4.026, *p* < 0.001, indicating that author names appear earlier in author lists when they have many versus fewer middle initials. In the secondary model, where we added covariates, the middle initial effect persisted, τ = -0.218, *S*_e_ = 0.059, *t*(1335.199) = 3.701, *p* < 0.001. In addition, a significant association between publication year and author position was obtained, τ = 0.013, *S*_e_ = 0.003, *t*(1316.673) = 4.832, *p* < 0.001, possibly reflecting increases in author amounts on articles over time. No significant associations were found between author position and gender, τ = 0.046, *S*_e_ = 0.060, *t*(1722.921) = 0.773, *p* = 0.439, and author position and impact factor, τ = -0.005, *S*_e_ = 0.015, *t*(1224.891) = 0.359, *p* = 0.720. The amount of authors yielded a significant association with authorship position, γ = 0.017, *S*_e_ = 0.003, *Z* = 6.834, *p* < 0.001, and a significant interaction emerged between the amount of authors in author lists and the amount of middle initials, γ = 0.006, *S*_e_ = 0.003, *Z* = 2.144, *p* = 0.032.

#### ‘At Least One versus None’ for All Articles

**FIGURE 2:**
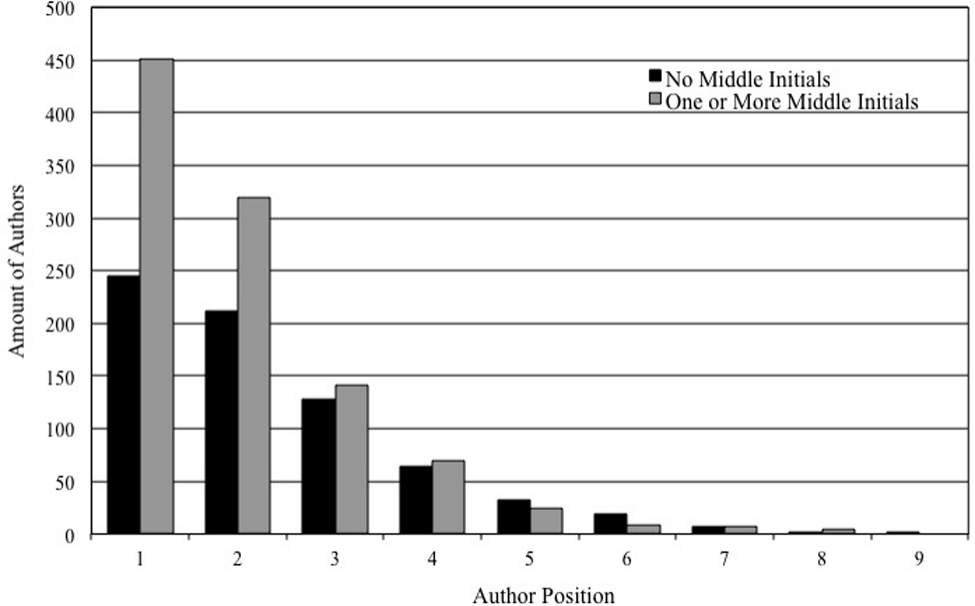
**The amount of authors for each author position based on the disaggregated data (Study 2)**.

Given that a large number of the authors had no middle initials in their names (40.9%), we conducted a similar multilevel analysis after recoding the amounts of middle initials into one group of authors with no middle initials, and one group of authors with one or more middle initials (see Study 1). Consistent with the previous results, the primary analysis indicated that the average position of authors with middle initials was significantly earlier in author lists (*M* = 2.00, SD = 1.23) than that of authors without middle initials (*M* = 2.34, SD = 1.43), τ = -0.309, *S*_e_ = 0.065, *t*(1681.334) = 4.755, *p* < 0.001 (for an overview, see **Figure 2**). Again, a significant middle initial effect was observed, τ = -0.258, *S*_e_ = 0.062, *t*(1517.921) = 4.144, *p* < 0.001, after controlling for the other variables in the secondary analysis. As before, a significant association between publication year and author position emerged, τ = 0.013, *S*_e_ = 0.003, *t*(1317.234) = 4.798, *p* < 0.001. No significant associations were found between author position and gender, τ = 0.047, *S*_e_ = 0.060, *t*(1723.637) = 0.787, *p* = 0.431, and between author position and impact factor, τ = -0.005, *S*_e_ = 0.014, *t*(1222.403) = 0.353, *p* = 0.724. The amount of article authors yielded a significant association with authorship position, γ = 0.017, *S*_e_ = 0.003, *Z* = 6.801, *p* < 0.001, and we found a marginal interaction between amount of authors on the articles and presence of middle initials, γ = 0.006, *S*_e_ = 0.003, *Z* = 1.909, *p* = 0.056.

#### ‘Many versus Fewer’ for Multi-Authored Articles

Consistent with the prediction, the first analysis revealed an association between authors’ amount of middle initials and their position in author lists, τ = -0.200, *S*_e_ = 0.065, *t*(1500.850) = 3.083, *p* = 0.002, indicating that author names appear earlier in author lists when they have many versus fewer middle initials. Controlling for the other predictors, in the secondary analysis, yielded a significant middle initial effect, τ = -0.180, *S*_e_ = 0.063, *t*(1117.852) = 2.838, *p* = 0.005. In addition, a significant association between publication year and author position was again obtained, τ = 0.010, *S*_e_ = 0.003, *t*(1114.096) = 3.294, *p* = 0.001. No significant associations were found between author position and gender, τ = 0.044, *S*_e_ = 0.065, *t*(1560.545) = 0.678, *p* = 0.498, and author position and impact factor, τ = -0.002, *S*_e_ = 0.016, *t*(1048.617) = 0.100, *p* = 0.921. The amount of article authors yielded a significant association with authorship position, γ = 0.014, *S*_e_ = 0.002, *Z* = 6.346, *p* < 0.001, and no significant interaction between amount of authors on the articles and amount of middle initials, γ = 0.004, *S*_e_ = 0.003, *Z* = 1.594, *p* = 0.111.

#### ‘At Least One versus None’ for Multi-Authored Articles

Again, we ran the multilevel analysis after recoding the amounts of middle initials into authors with versus without middle initials. Consistently, the primary analysis confirmed that positions of author names with middle initials appeared significantly earlier in author lists (*M* = 2.13, SD = 1.25) than those of authors without middle initials (*M* = 2.42, SD = 1.43), τ = -0.267, *S*_e_ = 0.069, *t*(1501.235) = 3.860, *p* < 0.001. Inclusion of the covariates still yielded the middle initial effect, τ = -0.228, *S*_e_ = 0.067, *t*(1338.260) = 3.416, *p* = 0.001. A significant association between publication year and author position emerged, τ = 0.010, *S*_e_ = 0.003, *t*(1115.877) = 3.240, *p* = 0.001. No significant associations were found between author position and gender, τ = 0.046, *S*_e_ = 0.064, *t*(1561.064) = 0.712, *p* = 0.477, and between author position and impact factor, τ = -0.001, *S*_e_ = 0.016, *t*(1047.529) = 0.089, *p* = 0.929. The amount of article authors yielded a significant association with authorship position, γ = 0.014, *S*_e_ = 0.002, *Z* = 6.307, *p* < 0.001, and we found no significant interaction between amount of authors on the articles and presence of middle initials, γ = 0.004, *S*_e_ = 0.003, *Z* = 1.368, *p* = 0.171.

#### Omissions of Middle Initials?

Again, we checked the data regarding potential omissions of middle initials for authors late in author lists using different sampling methods. For the first check, we drew two sub-samples with one author for each year from 1970 through 2013 (44 author names), resulting in an overall sample of 88 author names. The author positions were selected randomly. We found three omission errors, two at the first author position and one at the third author position. For the second check, we drew the author names for every third article of the 696 articles from 1970 to 2013 in the data set (i.e., 1/3 of the data set). Overall, we found only 10 omission errors, corresponding to less than 0.2% of the author names. Two of them were omissions at the first author position, five at second author position, and one omission error at the fourth and fifth author positions. These low numbers of omission errors and the relatively equal distribution across authors early and late in author lists indicate that the association between middle initials and author positions is unlikely explained by middle initial omission. Thus, it seems improbable that the reported middle initial effect is based on errors in reporting middle initials of authors later in the author list^4^.

The results based on articles from 1970 through 2013 across eight sub-disciplines in psychology indicate that author names with middle initials appear earlier in author lists of published articles than those without middle initials. This association emerged with or without covariates, whether including middle initials as continuous or dichotomous variable, and when analyzing all articles or only multi-authored ones. These findings are consistent with those of Study 1.

## General Discussion

According to American Psychology Association [APA] (2010) guidelines, authorship order should be decided based on contribution. Authors who contribute more should appear earlier in the author list. Early (vs. late) appearance in the authorship order of articles thus means that more (vs. less) ‘credit’ is given to authors for their contribution to the intellectual endeavor of conducting and publishing the research. There is no reason to doubt that academics in psychology consider the APA guideline when determining authorship orders. However, besides objective contribution, a range of other factors may subtly influence authorship orders (e.g., Fine and Kurdek, 1993; Thompson, 1994).

Based on the recent prior research on the middle initial effect (Van Tilburg and Igou, 2014), we wondered whether middle name initials in author names influence authorship positions. Given that middle initials affect people’s attributions of intellectual performance, we reasoned that middle initials in author names might influence the perception of intellectual performance associated with the research and publication process, which may then be reflected in the authorship order of journal articles in psychology. Based on this idea, we expected that authors with many middle initials appear earlier in author lists than authors with fewer middle initials, and that author names with at least one middle initial appear earlier in author lists than authors without middle initials.

To examine the association between middle initials and authorship order, we conducted two studies with large samples of academic journal articles published between 1970 and 2013 from overall 11 psychology journals. In Study 1, we examined articles from three prominent social psychology journals, and in Study 2, we examined articles from eight journals relating to different sub-disciplines in psychology. Taken together, our data included 1,374 articles and 3,860 authors.

We found that as the amount of middle initials increased, authors were more likely to hold the more honorable early positions in author lists than authors with fewer middle initials, and having no middle initial was least honorable in this regard. Middle initials in names were overrepresented for early positions in author lists.

In both studies, we employed multiple statistical tests to ensure robustness of the middle initial effect. First of all, we conducted two analyses of increasing complexity: In a first analysis, we examined whether middle initials were associated with author positions without any covariates. In a second analysis, we added covariates to test the robustness of this association: gender, year of publication, and impact factor. These covariates represent variables that are of potential interest when it comes to publication records (i.e., gender effects, robustness across time, standing of journal). Then, we repeated these two analyses after dichotomizing middle initials. Next, we performed all analyses again for only multi-authored articles. The different analyses are all important tests, from different angles. Every time the results were similar: The amount of middle initials was significantly negatively associated with author position. In essence, we included a range of analyses of different complexity to demonstrate the robustness of the association between middle initials and authorship order across specific models and analyses.

We also checked for potential mistakes in the reporting of names. However, there was very little evidence for omissions of middle initials, and they were not systematically related to author positions. We do not rule out that errors in reporting middle initials occur in the publication process, but it seems highly unlikely that such occasional errors explain the relationship between middle initials and authorship order.

### Author Positions of Junior versus Senior Academics

Many academics will be familiar with a more or less informal guideline that junior academics should ‘go first’ in the authorship order. This rule may be based on the observation that young scholars are often first authors on publications of, for example, their master thesis or dissertation or post-doctoral work. In a sense, this could reflect the APA guideline that authors who contributed most to the research project should appear early the authorship order. This guideline is not at odds with the middle initial effect that we report. Rather, a range of cues indicating responsibility and performance may affect authorship order.

If junior academics appeared often early in the authorship order *and* used their middle initials more than senior academics (e.g., to set themselves apart from seniors with similar names), then this could explain the reported middle initial effect. We have reasons to believe, however, that this is not the case. How could that work given that senior academics were once junior academics? Would they omit their middle initials over time as they become more senior? Given that omissions of middle initials do not explain the middle initial effect of authorship order, this seems unlikely. Maybe nowadays junior academics increasingly use their middle initials due to the larger number of similar author names in the field. If this were true, then the middle initial effect should be associated with the publication year. However, our analyses show that the middle initial effect was present when controlling for publication year, which in case of Study 2 ranged from 1970 to 2013.

As in some academic domains last author positions reflect seniority and prestige, one may wonder about association between last authorship and middle initials in author names. Importantly, supplementary analyses indicate that the amount of middle initials was not higher for last author positions, and the association between middle initials and authorship position remained significant when controlling for whether or not authors were listed last^5^.

### Middle Initials Matter in Academia

Our research explored an association between middle initials of authors and a performance indicator in academia. Specifically, this research addresses academic decisions that are directed at symbolizing intellectual performance via authorship order of academic journal articles. Academics may feel somewhat uncomfortable about the association between middle initials and authorship order of academic articles. Therefore, we assume that this research could spark discussions around the issue. To be clear, we believe that academic psychologists *do* consider APA guidelines. And we do not believe that editors or reviewers willingly evaluate journal manuscripts as a function of middle initials. We suggest that despite or given the complexity of the processes involved, a simple cue, namely the display of middle initials, may affect authorship orders. Further, we have no reason to believe that middle initials are diagnostic cues of intellectual performance in academia. It is unlikely that after thorough training and socialization into academia people with versus without middle names differ in their performance. In addition, it seems unlikely that the display of middle initials (i.e., whether someone reports a middle initial) is correlated with actual intellectual performance.

As it stands, our studies seem to point to a potential bias in academic decision making, consistent with the earlier results that middle initials lead people to infer greater quality of an essay than no middle initials (Van Tilburg and Igou, 2014). Given that authorship order symbolically represents achievement, it should be unbiased. However, when personal cues such as names of academics could impact on success, then this should make academics reflect about inferences regarding achievements based on such cues. It does not seem fair that academics with middle initials could have an advantage over academics without middle initials. We thus hope that our findings raise further discussions about the habits and fairness of measuring performance in academia (e.g., Fine and Kurdek, 1993; Thompson, 1994).

### Academia as a Real Life Context

The current research is an extension and application of the documented middle initial effect on status, and intellectual capacity and performance (Van Tilburg and Igou, 2014). In that research, a series of studies demonstrated the middle initial effect and examined boundary conditions and the inferential processes. Most directly related to the current research were the findings that writing performance of an academic extract was affected by the presence (vs. absence) of the authors’ middle initials (Study 1), and that the appearance of middle initials (Study 2) as well as their effect (Study 3) were bound to domains in which intellectual performance matter. The current research explored whether middle initials have an association to variables in a real life intellectual context, where inferences about performances matter: authorship order of academic journal articles. Our findings point to the importance of middle initials in this real life context.

### Limitations and Future Directions

Despite the interesting results of this research, we are aware of various limitations of these studies. Below we will discuss several limitations and flesh out potential future directions of research on authorship order as a function of middle initials.

#### Publications in Psychology

We focused on the association between middle initials and authorship order of articles in psychology. We made this decision because APA guidelines link authorship order to contributions in a relatively clear way. Norms and practices can be different in other academic disciplines (e.g., Einav and Yariv, 2006). Perhaps, authors with middle initials may be more likely to appear at positions that symbolize intellectual performance than authors without middle initials. However, the specific location of these would depend on the practices of the academic domains in question. Possibly, a last author position symbolizes intellectual performance more than earlier positions in some domains, leading to the hypothesis that middle initials would be overrepresented at the last author position.

#### Western Culture as a Limitation

Given that middle initials represent middle names, these cues are less likely to be influential in cultures in which middle names do either not exist or are perceived as unrelated to formal, intellectual contexts. We would not expect an association between middle initials and authorship order for academic journals where the display of middle initials is either unlikely or meaningless.

#### Correlations and Explanations

Earlier research (Van Tilburg and Igou, 2014) experimentally demonstrated that written extracts were evaluated more favorably if authors had names with middle initials than if they did not (Study 1). Further, the middle initial effect was limited to intellectual contexts (Studies 2 and 3). These experimental findings led us to design the current, correlative research, which in itself comes with limitations: The correlational work reported here does not demonstrate causality.

We concede that our studies did not directly test the specific psychological processes that may explain the asymmetry of middle initials and authorship order. The research was inspired by our earlier experimental findings that middle initials influence judgments and decisions because they symbolize status, intellectual capacity and performance (Van Tilburg and Igou, 2014). Our current results are consistent with this notion; however, a variety of processes may account for the association that we report. More specifically, based on our earlier work we reasoned that authors with middle initials and their work may be evaluated more favorably in terms of their potential and actual intellectual contributions to the research and publication process, thus influencing decisions on authorship order. For example, authors with middle initials may be given more responsibility in the research process or in the publication process because of the inferences that co-authors make about their capacity and performance. It may also be possible that during the publication process manuscripts first and second authored by academics with middle initials are evaluated more positively by reviewers and editors than other manuscripts, influencing the chances of publication. These and other hypotheses may explain the reported asymmetry that authors with middle initials appear earlier in author lists of academic articles, however, our studies do not indicate which specific processes may account for the association. Future research needs to examine the research and publication stages more closely, compare them, and control for actual intellectual contributions.

We were able to rule out potential influence of a number of variables regarding the association between middle initials and authorship order. Importantly, omission of middle initials belonging to authors who appeared later in author lists were rare and unlikely explained the observed associations. Further, although publication practices have changed over the years to some degree (e.g., increase in co-authors over the last decades), we observed the middle initial effect across several decades, which suggests that the effect is unlikely to be explained by cohort specific displays of middle initials over these years. Given that we used real world correlational data that was limited to publication records, it is possible that additional, at this stage not included, variables further explain the relationship between these name features and authorship order. Essentially, future research should test which specific processes, consistent or inconsistent with the notion of middle initial effects, account for the association between authorship order and middle initials.

#### Practical Implications

Assuming that middle initials indeed impact on authorship decisions at different stages, a number of measures may reduce this potential bias. These measures may be general ones that are designed to increase fairness of authorship order for multi-authored publications (e.g., Smith and Williams-Jones, 2012). Importantly, collaborators should communicate with each other about the project, authorship, and authorship order (see Strange, 2008), follow explicit rules (e.g., Gaeta, 1999), and clarify contributions via classifications (e.g., primary author, contributing author; see Baerlocher et al., 2007). Authorship orders could also be detached from contribution, similar to procedures in some academic domains (e.g., alphabetical orders; Einav and Yariv, 2006). Such name effects on publications may lead to requests for double-blind peer reviewing. Possibly, editors should also be blind to author names until after the editorial decision is made.

## Conclusion

Names matter to people (e.g., Nuttin, 1985, 1987), and they seem to affect life and death (e.g., Christenfeld et al., 1999; Einav and Yariv, 2006; Efthyvoulou, 2008). Our research suggests that middle initials are associated with authorship orders, one symbolic representation of intellectual performance, in academic journal articles in psychology. Authors with middle initials are more likely to be listed early in the authorship order, and authors with many middle initials have this advantage over authors with fewer middle initials. We hope our research instigates further discussions and additional research on the subject matter that address fairness in symbolizing and assessing intellectual performance via authorship order (e.g., Fine and Kurdek, 1993; Thompson, 1994).

## Conflict of Interest Statement

The authors declare that the research was conducted in the absence of any commercial or financial relationships that could be construed as a potential conflict of interest.

## Footnotes

1 We decided to first examine authorship orders in social psychology journals given the social psychological nature of this research: *Personality and Social Psychology Bulletin*, the *Journal of Personality and Social Psychology*, and the *European Journal of Social Psychology*.

2 We chose not to present additional statistical tests as the results were much the same as the original tests. Details can be provided on request.

3 We examined articles from psychology journals from a variety of sub-disciplines, excluding social psychology journals: *British Journal of Psychology*, *Cognitive Psychology*, *Developmental Psychology*, *Journal of Abnormal Psychology*, *Journal of Applied Psychology*, *Journal of Clinical Psychology*, *Journal of Personality*, and the *Journal of Psychology: Interdisciplinary and Applied* (formerly known as the *Journal of Psychology*).

4 We chose not to present additional statistical tests as the results were much the same as the original tests. Details can be provided on request.

5 For Study 1, when considering all articles, there was no significant difference in the amount of middle initials for last authors versus non-last authors, τ = -0.026, *S*_e_ = 0.022, *t*(1576.870) = 1.166, *p* = 0.244. Also, the association between middle initials and authorship position remained significantly negative after controlling for whether or not authors were listed last, τ = -0.152, *S*_e_ = 0.049, *t*(2112.746) = 3.118, *p* = 0.002. For Study 2, similar results emerged. There was no significant difference in the amount of middle initials for last authors versus non-last authors, τ = 0.027, *S*_e_ = 0.022, *t*(1329.721) = 1.230, *p* = 0.219, and the association between middle initials and authorship position remained significantly negative after controlling for whether or not authors were listed last, τ = -0.254, *S*_e_ = 0.056, *t*(1726.846) = 4.520, *p* < 0.001.
